# Immune Gene Rearrangements: Unique Signatures for Tracing Physiological Lymphocytes and Leukemic Cells

**DOI:** 10.3390/genes12070979

**Published:** 2021-06-27

**Authors:** Michaela Kotrova, Nikos Darzentas, Christiane Pott, Claudia D. Baldus, Monika Brüggemann

**Affiliations:** 1 Unit for Hematological Diagnostics, Department of Internal Medicine II, University Medical Center Schleswig-Holstein, Campus Kiel, 24105 Kiel, Germany; m.kotrova@med2.uni-kiel.de (M.K.); nikos.darzentas@gmail.com (N.D.); c.pott@med2.uni-kiel.de (C.P.); 2 Department of Internal Medicine II, University Medical Center Schleswig-Holstein, Campus Kiel, 24105 Kiel, Germany; Claudia.Baldus@uksh.de

**Keywords:** minimal residual disease, IG/TR rearrangements, real-time quantitative PCR, next generation sequencing, digital droplet PCR

## Abstract

The tremendous diversity of the human immune repertoire, fundamental for the defense against highly heterogeneous pathogens, is based on the ingenious mechanism of immune gene rearrangements. Rearranged immune genes encoding the immunoglobulins and T-cell receptors and thus determining each lymphocyte’s antigen specificity are very valuable molecular markers for tracing malignant or physiological lymphocytes. One of their most significant applications is tracking residual leukemic cells in patients with lymphoid malignancies. This so called ‘minimal residual disease’ (MRD) has been shown to be the most important prognostic factor across various leukemia subtypes and has therefore been given enormous attention. Despite the current rapid development of the molecular methods, the classical real-time PCR based approach is still being regarded as the standard method for molecular MRD detection due to the cumbersome standardization of the novel approaches currently in progress within the EuroMRD and EuroClonality NGS Consortia. Each of the molecular methods, however, poses certain benefits and it is therefore expectable that none of the methods for MRD detection will clearly prevail over the others in the near future.

## 1. Immunoglobulin and T-Cell Receptor Rearrangements

The remarkable ability of the human immune system to recognize and eradicate the enormous number of various antigens is based on the immune receptors [1]; the surface of T-lymphocytes is covered with the T-cell receptors (TR; TRαβ or TRγδ) and B-lymphocytes produce secreted or surface-bound immunoglobulins (IG). The tremendous diversity of the immune receptor variable domains is crucial for the specific molecular recognition of virtually any antigen [2]. Such a high degree of diversity is generated by combination of a limited number of gene segments. During this so-called somatic recombination, a DNA sequence that is unique for each lymphocyte is produced [3]. Hence, each lymphocyte bears many copies of the antigen receptor with a unique variable region that determines its antigen specificity.

### 1.1. Structure of the Immune Receptors

Both surface-bound and secreted immunoglobulins consist of two heavy chains (IGH) and two light chains (IGκ or IGλ), which are connected by a disulphide bond. The *IGH* gene complex consists of V (“variable”) segments at the 5’-end, which are followed by a group of D (“diversity”) segments and 6 short J (“joining”) segments. The gene segments for the constant (C) part of the heavy chain are localized at the 3′-end of the gene complex and are responsible for the immunoglobulin class determination. IG light chains are either encoded by a kappa (*IGK*) or by a lambda (*IGL*) rearrangement. The structure of these complexes resembles the structure of the *IGH* rearrangements but is composed of less V and J segments and does not contain any D segments.

TR molecules are also composed of two chains, connected by a disulphide bond. The “classical” type of TR contains (TRα) and (TRβ) chains, whereas the “alternative” type contains (TRγ) and (TRδ) chains [4,5]. The variable domains of TRβ and TRδ contain all three types of gene segments (V, D, and J). TRα and TRγ chains lack D segments, similarly to immunoglobulin light chains. The gene complex for TRδ is localized within the gene complex for TRα (between the V and J segments). Any V-J rearrangement of the *TRA* gene segments therefore results in the loss of *TRD* gene segments, which means that complete *TRA* and *TRD* gene rearrangements can never be present on a single allele simultaneously (with the exception of combined *TRDV-TRAJ* rearrangements [6]).

### 1.2. Somatic (V-D-J) Recombination

Somatic recombination occurs in immature lymphocytes in primary lymphoid organs (bone marrow for B-lymphocytes, thymus for T lymphocytes). V, D, and J gene segments of IG and TR genes are rearranged and a DNA sequence, which is unique for each lymphocyte, is produced. V-D-J recombination of *IGH* and *TRB* genes is a two-step process starting with a D-J recombination followed by a V-D recombination. The recombination of the *TRD* locus starts with D-D recombination and continues with V-D and D-J recombinations. Genes for Igκ, Igλ, TRα, and TRγ are produced by a one-step V-J recombination. During each recombination a random number of gene segments is excised and the remaining segments are joined together. This gives rise to tens of millions of possible combinations of V, (D) and J segments.

This process occurs in parallel on both chromosomes. As soon as a productive rearrangement is formed, it is transcribed into mRNA. At that point, the recombination process on the second chromosome is stopped and therefore only one type of antigen receptor is produced by each lymphocyte (allelic exclusion) [7,8].

### 1.3. Junctional Diversity

The joining of V, D, and J gene segments is a very imprecise procedure: the ends of germline segments that are being joined are cleaved randomly [3]. Moreover, the enzyme terminal deoxynucleotidyl transferase randomly adds so called “non-templated nucleotides” (N-bases) at the junctions between the gene segments that are joined together.

These sequence-altering processes further and vastly increase the diversity of antigen receptors and their ability to recognize virtually all possible antigens.

### 1.4. Affinity Maturation

Naïve B-cells express unmutated IG genes, but after the recognition of antigen by B-lymphocytes in secondary lymphoid organs (lymph nodes), an enzyme called activation-induced cytidine deaminase introduces somatic hypermutations around the productively rearranged V(D)J junction and cells with higher affinity for the antigen are favoured [9,10,11]. This event, called affinity maturation, enables the production of immune receptors with very high affinity to the certain antigen [12].

### 1.5. Rearrangement Process during Lymphocyte Development

The regulation of the V(D)J recombination in B- and T-cells is accomplished by different accessibility of IG and TR gene due to cell-type specific chromatin structures [13,14]. The IGH D-J joining starts in the ‘common lymphoid progenitor’ stage and *IGH* V-D-J, *IGK* and *IGL* rearrangements are initiated in the pro-B cell compartment. Rearrangement of the *IGK* locus either leads to IgH/κ expression or is followed by *IGK* deletion and *IGL* rearrangement, potentially leading to IgH/λ expression. The successful assembly of IG genes plays a crucial role in guiding B-cell development: B-cells lacking the capacity to rearrange their IG genes are arrested in pro-B cell stage, but the introduction of a rearranged *IgH* transgene allows the cells to progress to a pre-B cell stage [15,16,17,18,19,20,21].

TR loci rearrange in a highly ordered way. D-D and V-D rearrangements of the *TRD* locus begin in a pro-T (DN1) stage, followed by D-J rearrangements of the *TRD* and V-J rearrangements of the *TRG* locus in pre/pro-T (DN2) stage [22,23]. This is either followed by TRγδ expression or by *TRB* rearrangement in the pre-T (DN3) stage (D-J rearrangement). The *TRB* rearrangement is completed in a pre-T (DN4) stage (V-D-J rearrangements) [24]. Lastly, *TRA* locus rearranges in a double-positive stage [23,25], which is potentially followed by TRαβ expression. Because the *TRD* locus is nested in the *TRA* locus (between *TRAV* and *TRAJ* gene segments), rearrangement of the *TRA* locus leads to the deletion of *TRD* genes. This mechanism ensures that a single cell can either express a TRαβ or TRγδ, but not both.

## 2. IG/TR Rearrangements in Leukemia

### 2.1. Leukemic Clones & Oligoclonality

The entire set of antigen receptors with different antigen specificities in one individual is called the immune repertoire. In humans it is believed to be 1011–1012 or higher [1,26]. Thanks to the above-described recombination process, each newly developed B- or T-lymphocyte carries a uniquely rearranged junctional region sequence coding its antigen receptors.

Lymphoid malignancies are clonal diseases. It is therefore commonly believed that all their cells are descendants of a single malignantly transformed B- or T-lymphocyte and that the entire malignant clone carries identical IG/TR V(D)J rearrangement(s). Consequently, the junctional region is considered as a ‘DNA fingerprint’ of each particular clone [27].

Despite the generally accepted monoclonal origin of acute lymphoblastic leukemia (ALL), already early studies using PCR and Southern blotting reported that up to 40% of the B-cell precursor ALL cases are oligoclonal at diagnosis with up to 9 leukemic rearrangements per patient [28,29,30,31,32]. More recently, highly sensitive modern techniques employing next-generation IG/TR sequencing provided evidence that the percentage of patients with oligoclonal IG/TR profiles and also the degree of oligoclonality might be considerably higher [33,34]. Interestingly, oligoclonality at diagnosis is present in 27% of T-ALL patients harboring a cross-lineage *IGH* rearrangement [35], but TR oligoclonality in T-ALL is rather rare [27].

An oligoclonal IG/TR rearrangements profile is a consequence of continuing rearrangement and secondary rearrangement processes via the active recombinase machinery in these immature lymphoid malignancies [27]. Besides, up to a quarter of CLL patients harbor multiple dominant productive *IGH* rearrangements [36,37]. Only one third of these cases exhibit two clonal populations with distinct immunophenotypes [38], but each productive *IGH* rearrangement corresponds to a different B-cell clone also in immunophenotypically monoclonal cases [39].

### 2.2. IG/TR Rearrangement Profiles in Leukemias

Leukemic transformation leads to a cell differentiation arrest, which has direct impact on the IG/TR gene rearrangement configuration. Therefore, rearrangements in leukemic cells differ from the physiological repertoire and distinct rearrangement profiles can be identified according to age at diagnosis and genetic aberrations [40,41,42].

B-cell precursor ALL results from a leukemic transformation of a lymphoid precursor at an early stage of B-cell differentiation and it is therefore not surprising that over 80% of both adult and pediatric cases carry an *IGH* rearrangement and around 40% of them carry an *IGK* rearrangement [43,44]. As might be expected following the same logic, over 90% of T-ALL cases harbor a *TRB* rearrangement [45], over 80% a *TRG* and almost 70% (adults) or 40% (children) a *TRD* rearrangement [43,44]. Exceptionally, however, there are also ALL cases with all IG/TR loci in germline configuration—those are most probably derived from very immature progenitor cells.

Although cross-lineage rearrangements have not been detected in human thymocytes and their frequency in B-cells is very low (<0.5%) [46,47], these so-called illegitimate rearrangements have been identified in leukemic cells besides the lineage-consistent rearrangements: *IGH* rearrangements in 22% of T-ALL cases [35] and TR rearrangements in 80–90% of patients with B-ALL [48,49]. Cross-lineage rearrangements in B-ALL have several special characteristics, compared to regular rearrangements: *TRB* rearrangements contain particularly the most downstream Vb gene segments and solely the Jb2 segment, *TRG* rearrangements involve Jg1 segments in 70% of cases, and 80% of *TRD* rearrangements are represented by incomplete Vd2-Dd3 or Dd2-Dd3 junctions, which are rare in T-cells [44,48,50,51,52]. *TRB* rearrangements are virtually absent in pro-B-ALL and in infants, and patients with complete *TRB* gene rearrangements show a more mature IG/TR profile (higher frequency of *IGK*, *TRG*, and Vd2–Ja rearrangements) [53]. Remarkably, the frequency of cross-lineage Vd2-Dd3 rearrangements significantly decreases with age at diagnosis, while cross-lineage *TRG* rearrangements are rarely found in patients below 2 years of age [27,41]. In T-ALL, the cross-lineage *IGH* rearrangements are rather immature, as they are characterized by a high frequency of incomplete D-J rearrangements and frequent usage of most downstream Dh6-19 and Dh7-27 and most upstream Jh1 and Jh2 gene segments [35]. Cross-lineage rearrangements are rare in mature B- and T-cell malignancies, probably due to the absence of recombinase activity [54,55,56]. This corresponds with the reported decreasing incidence of cross-lineage *TRG* rearrangements in more mature B-ALLs: pro-B (57%), common (47%), pre-B (42%), and mature-B (0%) ALL [49]. Also, in more mature T-ALLs with biallelic *TRD* deletions and completed *TRA* rearrangements the *IGH* gene rearrangements are virtually absent [35]. In contrast to CLL, a mature B-cell malignancy, high incidence of non-coding/out-of-frame rearrangements was observed in ALL, suggesting that antigen selection pressure does not play a crucial role in ALL [57].

In CLL, so called stereotyped B-cell receptors are a common phenomenon. Their complementarity-determining region 3 (CDR3) sequences are closely similar (share structural features like V-gene, length, amino acid composition) among unrelated cases, suggesting that stimulation by (auto)antigens may play a role in CLL pathogenesis [58,59]. The *IGH*/*IGK*/*IGL* repertoires in CLL are biased and differ from repertoires in normal B-cells [60,61,62,63]. Additionally, certain V-segments (*IGHV3-21* and *IGHV1-69*) are associated with poor outcome [57]. Furthermore, presence of somatic mutations in variable heavy chain genes defines two CLL subtypes associated with a different clinical course. About half of CLL cases have more than 98% identity to the closest germline V-gene (“unmutated”), which corresponds to inferior outcome compared to patients with “mutated” CLL (less than 98% identity) [64,65].

### 2.3. Stability and Sensitivity of IG/TR Rearrangements as MRD Targets

Since IG/TR rearrangements are not directly related to the oncogenic process, they may vanish over time due to the outgrowth of subclones or ongoing and secondary rearrangements in leukemic blasts with active IG/TR recombination machinery. This might lead to an underestimation of MRD level if a rearrangement is only present in a small subclone, or even a false-negative MRD result if the rearrangement is fully lost during the disease course. It has therefore been recommended to use at least two leukemia-specific rearrangements to detect MRD in ALL to lower the risk of obtaining a false negative result [66].

Studies comparing the rearrangement profiles at diagnosis and during the disease course or at relapse are almost exclusively focusing on pediatric patients. It has been shown that oligoclonality at diagnosis is the most powerful predictor of ongoing clonal evolution in ALL: particularly in childhood BCP-ALL, significant differences in stability were observed between monoclonal and oligoclonal rearrangements: 89% of monoclonal vs. 40% of oligoclonal rearrangements are preserved at relapse [67]. In this study, roughly 85% of monoclonal *IGH* and *TRD* rearrangements remained stable between diagnosis and relapse. Among monoclonal IGK-Kde rearrangements the percentage of stable targets is even higher (95%), probably due to their end-stage character [68]. A study comparing IG/TR profiles at diagnosis and relapse of B-ALL employing high throughput IG/TR sequencing confirmed that the overall stability of IG/TR rearrangements is rather low in (27% of clonal rearrangements were preserved), but also showed that the stability of large clones is way higher (84%) [69]. At relapse, the general characteristics of the IG/TR gene profiles are comparable to those at diagnosis but exhibit a lower degree of oligoclonality and more frequent *TRD* gene deletions, which fits with the hypothesis of ongoing clonal selection and continuing rearrangements [67]. In T-ALL, the IG/TR rearrangements profiles at diagnosis and relapse are more stable: 97% and 86% of TR rearrangements are preserved at relapse in adult and childhood T-ALL, respectively [70]. *TRD* rearrangements are the most stable ones (100% of rearrangements preserved at relapse), followed by *TRG* (89%) and *TRB* rearrangements (82%) [70].

Besides different stability during the disease course, IG/TR MRD targets also vary in sensitivity of the derived real-time quantitative polymerase chain reaction (RQ-PCR) assays. The sensitivity is primarily determined by the combinatorial and junctional diversity of the CDR3 regions. Therefore, RQ-PCR assays based on rearrangements from IG/TR loci that contain more V/D/J gene segments in their germline sequence (higher combinatorial diversity) generally have higher sensitivity. Similarly, complete rearrangements that contain D-segments (*IGH*, *TRB*, *TRD* V-D-J rearrangements) provide higher sensitivity than complete rearrangements without a D-segment (*TRG*, *IGK* rearrangements) and incomplete rearrangements. For example, complete *IGH* rearrangements represent the most sensitive group of targets, usually reaching the sensitivity of 10^−4^ [43]. Also, complete *TRB* rearrangements provide decent sensitivity thanks to their extensive junctional regions [70]. The lower combinatorial diversity in incomplete *TRB* rearrangements provides an explanation for slightly lower sensitivities in this group of targets [53]. In contrast, the sensitivity of *TRG* targets is usually considerably limited (a sensitivity of at least 10^−4^ is reached in less than half of the patients), owing to the restricted size of their junctional regions and the non-specific amplification of highly abundant polyclonal *TRG* rearrangements in normal T-cells [71]. Intriguingly, *TRG* rearrangements contain significantly higher number of inserted nucleotides and lower number of deleted nucleotides in T-ALL than in BCP-ALL, which seems to be the most important predictor for reaching good sensitivity [71].

## 3. Minimal Residual Disease

### 3.1. MRD as an Important Prognostic Factor in Lymphoid Malignancies

Besides conventional patient-associated (e.g., sex, age at diagnosis, ethnicity) and disease-associated (e.g., white blood cell count at diagnosis, immunophenotype, genetic aberrations) prognostic factors, MRD after induction/consolidation therapy reflecting the treatment efficiency has proven to be most important in predicting patient outcome and risk of relapse in ALL [72,73,74,75,76,77,78,79,80,81,82,83]. The significance of MRD for prognostication has increased over the last decades also in CLL and multiple myeloma. MRD status after the induction phase of treatment is highly significant for predicting (relapse-free) survival, according to several large trials [84,85,86,87,88,89,90,91,92,93,94].

MRD is therefore widely used to identify high risk ALL patients who benefit from treatment intensification and, on the other hand, patients in whom treatment de-escalation, reducing treatment-related toxicity, is feasible. MRD is also monitored in the vast majority of clinical trials, where reaching MRD negativity is considered an important endpoint for the determination of treatment efficiency.

### 3.2. MRD Detection Techniques

Both low- and high-throughput methods for molecular detection of MRD in lymphoid malignancies either utilize IG/TR rearrangements or fusion gene transcripts associated with the leukemia. IG/TR rearrangements can be detected in 95% [32] of ALL patients and in virtually all CLL patients. Fusion gene transcripts can be detected only in 40–45% of patients with B-ALL and 15–35% of patients with T-ALL [95].

Current standard for molecular MRD detection is an IG/TR-based RQ-PCR, a low-throughput method which has been extensively optimized and standardized within the EuroMRD (www.euromrd.org, accessed on 26 June 2021) Consortium. The sensitivity routinely reaches 10^−4^ to 10^−5^ (1 leukemic cell in 10,000 to 100,000 healthy cells). Because each patient’s leukemia-specific IG/TR rearrangements are different, the assay characteristics (sensitivity and the quantitative range) differ among patients and even within a single patient. Therefore, the sensitivity and the quantitative range of IG/TR-based RQ-PCR has to be assessed separately for each individual assay. This is not the case of MRD assays employing fusion gene transcripts since those are shared among patients and no patient-specific assays are therefore necessary. These assays generally reach a sensitivity of 10^−5^.

Another low-throughput method that can be used for MRD detection is digital droplet PCR (ddPCR). This method builds on the workflow of RQ-PCR. However, thanks to compartmentalization of the reaction into oil droplets and the resulting digital readout, absolute quantification of target DNA without the need of a reference standard curve is possible.

The laboriousness and tardiness associated with the necessity of using patient-specific assays if IG/TR rearrangements are used as markers, can be overcome by next-generation sequencing (NGS). Unlike Sanger sequencing, which provides one representative sequence for all molecules in the PCR, the high throughput of next-generation sequencers allows for obtaining the sequence information for each molecule separately. Using this technique, the same primer set(s) can be used for all patients.

Residual leukemic cells can also be detected by multiparameter flow cytometry (MFC) which can identify cells with leukemia-associated immunophenotype (LAIP), differing from that of normal cells. This approach is way faster compared with the above-mentioned molecular methods and is applicable in >98% of patients with ALL and all patients with CLL [96,97]. The sensitivity of flow cytometry can reach the levels obtained by the molecular methods, provided that a sufficient number of cells are evaluated [97].

### 3.3. IG/TR-Based MRD Detection by Low and High Throughput Molecular Methods

**Figure 1:**
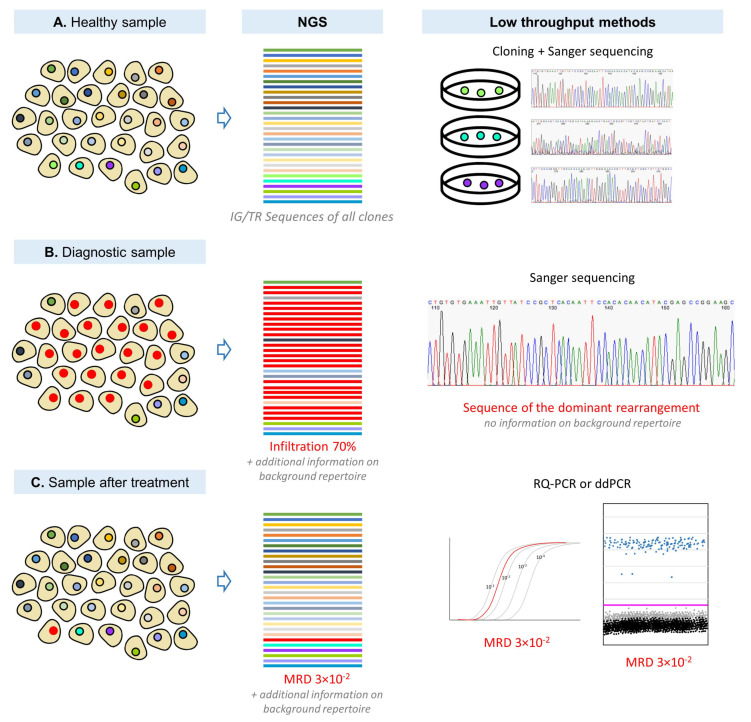
Low- and high-throughput (NGS) IG/TR analyses in different sample types. (**A**) In samples from healthy individuals, NGS provides sequences of all clones. These can also be obtained by low-throughput Sanger sequencing, but it is necessary to clone the PCR products before sequencing to obtain a clean sequence of each clone separately. (**B**) In highly infiltrated diagnostic samples, NGS provides a sequence of the dominant malignant clone (red) and background clones, as well as information on their abundance. Sanger sequencing provides a sequence of the dominant rearrangement only, without abundance information. (**C**) In samples after treatment, NGS can detect the rearrangement of the malignant clone (red) and the background rearrangements. RQ-PCR or ddPCR only provide the information about the MRD level.

Regardless of the molecular method used for IG/TR-based MRD detection, characterization of IG/TR rearrangement profile and identification of leukemia-associated IG/TR rearrangement(s) must be performed in each diagnostic sample with a panel of screening PCRs followed by Sanger sequencing or NGS of clonal PCR products (Figure 1). Once the sequence of the leukemia-associated rearrangement(s) is acquired, it can be used as a marker for the detection of MRD.

**Figure 2:**
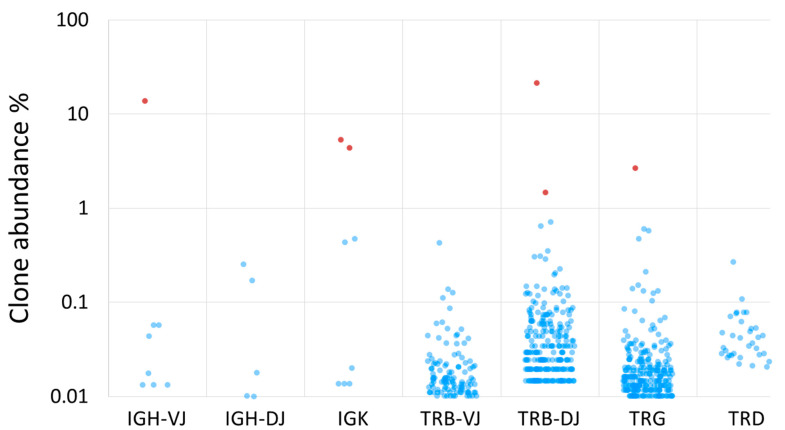
Normalized abundances of IG/TR clones in the diagnostic sample of a patient with B-cell precursor ALL. The sample was analysed using the EuroClonality-NGS assays [99]. Clone abundances were normalized using the EuroClonality-NGS spike-in controls [107]. Clones with an abundance <0.01% are not shown. Leukemia-associated clones which can be used for MRD-detection are shown as red dots, background physiological rearrangements as blue dots.

Until recently, multiplex assays developed in a European BIOMED-2 collaborative study [98] and low-throughput Sanger sequencing have been commonly used by most laboratories. However, with the advent of NGS, new assays for amplicon NGS have been developed by the EuroClonality-NGS working group (https://euroclonalityngs.org/, accessed on 26 July 2021) [99], which is formed by several European groups with expertise in assay design, immunobiology, bioinformatics and clinical application of IG/TR analysis in hemato-oncology, pathology, and immunology. Improved primer sets for use with NGS were designed for a better coverage and equal amplification efficiency of all rearrangements. NGS-based approaches perform better in samples containing bi-allelic rearrangements, polyclonal background and in oligoclonal samples, where the low-throughput sequencing provides mixed sequences that cannot be used for patient-specific primer design [99] (Figure 2). The NGS-based marker identification has already been introduced in many MRD laboratories and the EuroMRD Consortium has already included NGS-based marker identification in its quality control rounds.

Despite current advances in the field of molecular MRD detection, RQ-PCR of clonal IG/TR gene rearrangements remains the most widely used molecular method for MRD assessment due to the lack of standardization of the modern methods. It requires junctional region-specific forward primer, reverse primer or probe (called allele-specific oligonucleotides (ASO)) designed based on the leukemia-associated sequence obtained during the initial marker screening. The most common strategy is the ‘ASO forward primer approach’, using germline probes and reverse primers located in the J gene segments. It has several advantages compared to the ‘ASO reverse primer’ and ‘ASO probe’ approaches: (1) because the number of J gene segments is lower than the number of V gene segments, lower number of germline probes and reverse primers is needed; (2) the binding sites of germline probes and primers located in the J-gene segments remain unaffected in cases with ongoing rearrangements, V replacements and somatic hypermutations. The sensitivity and quantitative range of each assay must be determined separately based on the performance of a dilution curve from the diagnostic material of each particular patient. These must be taken into account for the final interpretation of RQ-PCR MRD results according to the international guidelines [100]. The need of a dilution curve can limit the applicability of the RQ-PCR in patients with insufficient amount of diagnostic DNA.

The patient-specific primers/probes are also indispensable for ddPCR-based MRD detection, therefore even in this setting, the laborious design of patient-specific assays cannot be avoided. ddPCR can however overcome certain limitations of IG/TR-based RQ-PCR and can therefore serve as an attractive low-throughput alternative for MRD detection. In particular, ddPCR provides an absolute MRD quantification without the need of a dilution curve. This is crucial for high-risk patients in whom repeated MRD monitoring is required, because the amount of diagnostic material necessary for the standard curve is limited. Still, no guidelines for ddPCR-based MRD detection have been defined so far and its prognostic power is still under investigation. The EuroMRD Consortium is currently putting great effort into standardization of the method for MRD detection in lymphoid malignancies.

On the contrary, no patient-specific oligonucleotides are needed for NGS-based MRD detection. The same multiplex primer set used for marker identification can be used for MRD detection in all patients, hereby avoiding the cumbersome design and testing of patient-specific ASO RQ-PCR assays. Although the wet-lab part of NGS-based MRD detection is more straightforward when compared to low throughput methods, the subsequent data analysis is challenging and requires expertise and specialized bioinformatics tools. As already mentioned above, NGS provides a sequence of each single IG/TR rearrangement in the tested sample. This is useful for detection of emerging leukemic (sub)clones resulting from the ongoing rearrangement activity and also assessing the background IG/TR repertoire. Nevertheless, as with the ddPCR, the standardization of the method and assessment of its prognostic power on large patient cohorts is still in progress within the EuroClonality-NGS working group.

Although NGS and ddPCR have not yet been fully implemented into routine practice, there are hints that both methods possess appreciable advantages over RQ-PCR. Particularly samples which are positive below the quantitative range (pos < QR) of RQ-PCR can often be assessed more confidently: thanks to the more specific readout of NGS, false-positive RQ-PCR results which might occur due to the nonspecific amplification can be reliably distinguished from truly positive samples [101]; ddPCR is able to provide an accurate MRD quantification in 20–45% of pos < QR samples thanks to its absolute quantification without the need of a standard curve [102,103,104,105]. Another frequently mentioned advantage of the modern methods is the improved sensitivity. However, such conclusions must be interpreted cautiously. The sensitivity, which is often demonstrated on artificially prepared MRD samples (serial dilutions of patient material/cell lines/plasmids in healthy mononuclear cells), may not necessarily reflect the reality in the clinical setting. Fundamental factors influencing the sensitivity are the number of analyzed cells (DNA amount) and the sequencing coverage. While increasing the sequencing coverage is only a matter of costs, the amount of valuable patient DNA is an inevitable limiting factor. One might assume that to reach a sensitivity of 1 in 1 million (10^−6^), it is sufficient to analyze DNA from a million cells (equivalent to roughly 6.5 μg). Nevertheless, several critical factors increase the requirements on the cell quantity significantly. Firstly, randomness of subsampling must be taken into account. According to the Poisson statistics, threefold amount of DNA (i.e., 20 μg for the sensitivity of 10^−6^) is necessary to confidently detect a single cell in 95% of cases [106]. Additionally, loss of material during numerous laboratory steps and an unequal amplification efficiency for different IG/TR rearrangements further increase the DNA quantity requirements.

## 4. Summary

Thanks to their uniqueness, IG/TR rearrangements can be used as strongly specific molecular markers of lymphocyte clones. Often being referred to as ‘molecular fingerprints’, virtually any physiological or malignant lymphocyte clone can be detected using IG/TR rearrangements as markers. Although modern molecular methods bring certain benefits, mainly in the form of higher specificity and possibly also additional information on the background clones, their standardization and validation in a clinical setting has not yet been finalized. Nonetheless, the modern methods are already being very useful in a research setting and in special cases where RQ-PCR does not provide unambiguous results. Whether any of the aforementioned molecular methods is used, the interpretation of the IG/TR data is a highly complex topic, requiring a high level of expertise and should therefore be only performed by well-trained professionals.
